# Notes and News

**Published:** 1887-03-12

**Authors:** 


### Notes and News.

COLLECTED AND COMMUNICATED.

WE remind our readers of an important meeting of the Hospitals Association on Wednesday next, when Mr. Samuel Benton will read a paper on "Nursing in Workhouse Infirmaries." Full details will be found on page ii of the advertisements.

DR. RAYNER, of Hanwell Asylum, will deliver a lecture upon "Insanity as related to Childbearing," at the Midwives' Institute, 15, Buckingham Street, Strand, on Friday, the 11th inst., and Dr. T. Dowse will lecture on the 25th inst., at the same place, on the subject of "Massage."

THE resolution recently passed by the delegates of the Hospital Saturday Fund, fixing the street collection this year for June 25, has been rescinded, and it has now been resolved to hold it on June 11th. Lieut.-Col. Charles Mercier and the Rev. J. R. Diggle, M.A., have been elected Vice-Presidents of the Hospital Saturday Fund.

THE total cost of the recent drainage works at the West London Hospital amounted to £1,588 12*s*. 6*d*. This has been a heavy tax upon an institution already somewhat deeply involved in debt, but a hospital, above all other places, should be perfect in its sanitation, and the money which has been spent under this heading has, we think, been wisely expended.

THE late M. Rimmel, the well-known perfumer, of the Strand, took a very warm interest in the French Hospital and Dispensary in Leicester Square. In conjunction with Dr. Vintras and one or two other gentlemen, he took a prominent part in the foundation of this institution just twenty years ago.

SIR T. SPENCER WELLS, Bart., F.R.C.S., at the fifth annual meeting of the Governors of the Hampstead Home Hospital, held at the Hampstead Public Library last Saturday, made a very just observation about nurses. In connection with this hospital, which is situated at Parliament Hill Road, Hampstead, there is a nursing institution for training ladies who desire to take up the occupation of nursing and for supplying the public with trained nurses. Last year the nurses sent out to attend private cases earned £110 19*s*. Sir T. Spencer Wells, in moving the adoption of the report, spoke in terms of high praise of the efficiency of the work performed by these nurses, and urged that they ought not to be treated by the families engaging them as domestic servants, but as ladies. The public generally have not grasped the fact that a vast change for the better has been wrought in the profession of the nurse; that it is one that is now adopted by well-educated women, and is becoming less and less the sphere of any ignorant person desirous of eking out a bare subsistence.

HOSPITAL and infirmary collecting-boxes appear to be prized by the light-fingered fraternity. Twice, quite recently, they have stolen a box belonging to the Kent and Canterbury Hospital from the Falcon Hotel, Canterbury, and now the Peterborough Infirmary collecting-box, at the Great Northern Railway Station, Peterborough, has once more been broken open and its contents stolen. A burglar-proof collecting-box is an invention which some ingenious mind might essay.

THE Deaconesses' Institution and Hospital at Tottenham has just received from Mr. Howard and Mr. Charles Morley (sons of the late Mr. Samuel Morley) the munificent gift of £4,500, the amount of the debt existing on the third wing which has recently been added to the hospital. We learn from Dr. Laseron, the director, that it is intended to name the new building the "Samuel Morley Wing."

THE Queen, in response to an appeal recently made to her from the East-end to beg her acquiescence in a movement to commemorate the Jubilee by a fund in support of the London hospitals, dispensaries, and convalescent homes, has replied that she is unable to express any special approval of the very numerous schemes which are proposed for celebrating her Jubilee, though she is gratified at the loyal feelings which prompt them.

THE one hundred and thirty-sixth annual report of the City of London Lying-in Hospital was read at the last General Court of Governors, and our readers will be glad to learn that to this old-established charity at all events the year 1886 has proved a fairly satisfactory one. During that period 283 in-patients had been received, and since May not one death had occurred in the hospital. The incubator (which useful invention has already been alluded to in our columns) proved of great service in helping to maintain animation in several apparently still-born children. The total income for the year, added to the balance last carried forward, was £3,166 11*s*. 1*d*., whilst the expenses were £2,925 3*s*. 9*d*., leaving a balance of more than £240 to the credit of the institution. A sum of £358 had been expended for painting and cleaning the hospital inside and out, and for building a new room over the chapel, and there will consequently be no occasion to suspend the work of the charity for repairs, etc., during the present year. Dr. Yarrow was unanimously elected surgeon-accoucheur, Mr. John Davies, the other candidate for the post, having retired in his favour.

---

The following vacancies are announced this week, the amount of the salary being placed in each case after the name of the institution :—

**Resident Medical Officers.**

Derby Infirmary. House Surgeon. Qualified.—£100.
East London Hospital, Shadwell. Clinical Assistant.—No salary.
Hastings Hospital. House Surgeon. Doubly qualified.—£70.
Lock Hospital, Soho. House Surgeon. Qualified.—£50.
Nottingham Hospital. One Medical, one Surgical Assistant. —No salary.
Stockport Infirmary. Assistant House Surgeon. Doubly qualified.—£70.
Westminster Western Dispensary. One. Doubly qualified.— £105.

**Non-resident Medical Officer.**

Holborn Union. Mitcham Workhouse. Qualified.—£100.

**Matrons.**

Bromyard Cottage Hospital. Nurse and Matron.
Devonport Royal Albert Hospital. One. £50.

**Trained Nurses.**

Exeter Hospital. Surgical Nurse.—£30.
Gloucester Children's Hospital. One; Certificated Monthly and Midwife.—£21 and uniform.
Isle of Wight Nursing Institution. One District, one General. —£22 and £27.
Newcastle Nurses' Home. One District.—£40.
Portsmouth Royal Hospital. One Night.—£20.
Weston-super-Mare Hospital. One.—£25.
Windsor Infirmary. One.—£20.

**Lady Probationer.**

Grantham Hospital. One.

---

The eleventh annual general meeting of the supporters of the Ashburton and Buckfastleigh Cottage Hospital was held on February 4th, the Rev. W. M. Birch presiding. The number of patients during the past twelvemonth was unusually large compared with preceding years, forty-six cases having been treated, and the average residence in the hospital of each sufferer was stated to be forty-one days. This somewhat extraordinary average was due to the fact that several cases were the result of severe accidents, and demanded much attention for many weeks. The receipts of the institution amounted to £227 12*s.* 5*d.*, and the expenditure to £225 2*s.* 11*d.*, leaving £2 9s. 6*d.* to be added to the balance of £17 8*s.* 1*d.* from the preceding year. Mr. Fabyan Amery, the Honorary Secretary, also read the report of a special committee which had been appointed to consider the question of erecting a new cottage hospital at Ashburton, for occupation when the lease of the present buildings expired. Upwards of £1,043 had been promised by supporters of the scheme, and half an acre of land in a favourable position had been offered as a free site by Mr. R. G. Abraham. The committee were therefore strongly in favour of the work being commenced without delay, and it was decided by the general meeting that steps to obtain plans and specifications should at once be taken. We may add, to show the desirability of maintaining such an institution, that Ashburton is twenty miles from any large county hospital.

---

The Burnley Hospital possesses features distinct from all other circular hospitals yet built. The two wards just opened are situated at the end of corridors leading from the administrative block, a plain three-storied structure, where accommodation for doctors, nurses, out-patients, cooking, washing, etc., is provided. On either side of a short passage leading from the corridor to the wards are two small isolation wards, nurses' room, and scullery. The ward itself is circular, or rather annular, as the central space is occupied by a shaft sixteen feet in diameter. There are twenty windows in each large ward, and between every two a bed is placed. The shaft in the middle contains a gently ascending spiral staircase, which winds round a central flue, containing the chimney, heating arrangements, and a shaft for the extraction of foul air. Ascending the staircase we come to a verandah, which opens into the convalescent room, situated on the centre of the roof of the ward, the sides of which are mostly of glass. The space outside the convalescent room, and which completely encircles it, has been converted into an open-air promenade twelve feet in width. Our readers will see from this description that there are many novelties of construction and arrangement to be studied and criticised.

---

The heating of the wards and of the administrative block, on the advice of Captain Douglas Galton, will be by steam. The pipes have been carefully graded to permit a free return of the condensed water, and by this means those distressing noises, so common where steam is used as a means of heating, are said to be avoided. A series of pipes runs round the ward and around the central shaft, so that the amount of heat can be regulated at will. Placed in the central flues are a series of steam coils connected by tubes with the ward. These steam coils, by raising the temperature of the air, cause an upward current, and the vitiated air from all parts of the ward is then carried off through the open tubes. The coils are four in number, and of different sizes. They are so arranged that the number employed, and consequently the extracting force used, can be easily regulated. The walls of the wards are lined with glazed bricks, the roof is cemented, and the floors are laid in polished oak. All the windows are of plate-glass. The building stands well, and is placed in three acres of ground which has been tastefully laid out. Altogether, it must be admitted that the Burnley Hospital marks a new departure in hospital construction, and no doubt many people will be attracted to the town by a desire to examine so novel and unconventional a building as we have here briefly described.

---

Messrs. Blaikie have recently erected at the Aberdeen City Hospital a very fine disinfector, made by Messrs. Manlove, Alliott & Co. of Nottingham. The apparatus, which is known as the Washington Lyon's Patent Steam Disinfector, uses no chemicals in the process of disinfection, and any article to be cleansed need not be unmade; colours, however delicate, are not materially affected, and manuscript documents are disinfected without injury. Moreover, the unpleasant smell which the use of carbolic acid entails, is entirely avoided. The principle involved in the invention is the destruction of the germs of disease by the introduction of superheated steam under regulated pressure into a specially constructed apparatus.

THE following sums have been recently received by the various Medical Charities, the name of the donor or the source from which they were obtained being given in each instance after the name of the Institution :—

**Donations.**

North London Hospital. Mrs. S. Vokes, £500. Mr. A. C. Barclay, £31 10*s*. Company of Leathersellers, £21.
Metropolitan Convalescent Institution, Walton-on-Thames. The Company of Leathersellers, £21 for General Fund ; £10 10*s*. for Seaside Branch. Miss Swinburne, £10.
Trained Nurses' Annuity Fund. Company of Clothworkers, £20. Company of Leathersellers, £10 10*s*.
London Temperance Hospital. T. E. H., £100.
National Hospital for Consumption, Ventnor. Mr. J. R. Allen, £31 10*s*.
London Fever Hospital. Mr. E. S. Barker, £10 10*s*. Mr. E. L. Cox, £10 10*s*. Mrs. McCalmont, £10 10*s*. Dowager Lady Wenlock, £10 10s.
Hospital for Diseases of the Throat, Golden Square. Messrs. Baring Bros. & Co., £10 10*s*.
Queen Charlotte's Lying-in Hospital, Marylebone Road. Mrs. Winans, £21.

**Legacies.**

King's College Hospital. Mr. G. W. P. Bentinck, £1,000.
St. Mary's, Paddington. Mr. G. W. P. Bentinck, £1,000.
Charing Cross Hospital. Mr. G. W. P. Bentinck. £1,000.
St. George's Hospital. Mr. G. W. P. Bentinck, £1,000.
Great Northern Hospital. Mr. G. W. P. Bentinck, £1,000.
University College Hospital. Mr. G. W. P. Bentinck, £1,000.
Hospital for Sick Children, Great Ormond Street, Mr. G. W. P. Bentinck, £1,000.
London Hospital. Mr. G. W. P. Bentinck, £1,0^0.
London Homœopathic Hospital, Great Ormond Street. Mr. G. W. P. Bentinck, £1,000.
British Home for Incurables. Miss M. J. Render, £19 19*s*.
North Eastern Hospital for Children, Hackney. The Trustees of Prison Charities, per Mr. John Watney, £21.
Bexhill-on-Sea Convalescent Institution. The Trustees of Prison Charities, per Mr. John Watney, £52 10*s*.

---

MR. WILLIAM EVANS, the Chairman of Committee of the Birmingham General Hospital, recently gave some very suggestive facts with regard to his institution. A century ago, the income of the General Hospital amounted to about £1,100 ; now it stands at £10,000. In 1785 there were 400 in-patients and 463 out-patients ; while last year there were 3,400 in-patients and nearly 38,000 out-patients. "If," remarked Mr. Evans, "the income of £1,100 was a fair and handsome income for 800 patients a century ago, the income of £10,000 in the present day is, I think, very inadequate when compared with the increase in the number of patients." Its income for many years past has not kept pace with the constantly increasing demands upon its resources, and the result is that the institution is now burdened with the incubus of a heavy debt. The growth of this debt has, by the exercise of a rigid economy, been stopped ; but, during the present year, a supreme effort ought to be made for its material reduction. It has been currently said in some quarters of Birmingham, that the General Hospital is a wealthy institution ; but the facts unfortunately point to a very different state of things. That little suburban hospital, which bears the honoured name of Jaffray, has, of course, somewhat drawn upon the funds of the parent institution, but the patients who have been drafted thither from the great building in Summer Lane, have mostly reaped material benefit, and the branch has, on the whole, proved a most valuable adjunct.

---

THE hospitals all over the country have shown a very praiseworthy activity in putting themselves *en évidence* thus early in the year of Jubilee. The enthusiasm which has been evoked on behalf of our charities wherever men have met together to discuss the manner in which they will celebrate the great event which is at hand, shows that the spirit of charity "slumbereth not." Never before have our hospitals aroused an interest at once so deep and so far-reaching as the Jubilee celebration is calling forth. Other schemes—almost without number—have, of course, found their advocates : free libraries, local improvements of various kinds, open spaces, galas, firework displays, etc., have been suggested, and in some instances have been adopted ; but it has been very noticeable that, where a hospital has figured in the programme, the other proposals have had to go to the wall. Indeed, the fear is that the Queen's Jubilee will have been the means in some quarters of multiplying our hospitals unwisely. When there are already institutions making a desperate struggle for existence, it is very undesirable to add others—unless, indeed, their endowment, as well as their erection, can be accomplished. The spirit, however, which is abroad is of the right kind, though it may require to be guided with caution and with wisdom. The signs of the times mark out the year of Jubilee as a turning point in the history of our charities, the *renaissance*, let us hope, of the hospitals throughout the land.

---

DURING the past nine years, some four hundred consumptive patients in New South Wales have at different times found a home at the expense of Mr. J. H. Goodlet of Sydney, whose noble philanthropy has made his name so well known throughout the colony. This good work has hitherto been carried on in a house near the Picton Railway Station, but is now to be transferred to a special building, about three miles distant from the old Home, on a site which has been given and endowed by Mr. Goodlet. It will accommodate forty-eight patients, twenty-four of each sex, and has also rooms specially devoted to the comfort of Mrs. Price, the matron, and her assistants, as well as sitting, dining, and smoking rooms, kitchens, etc. We are rejoiced to hear that the traditions of English philanthropy are so well maintained amongst our fellow countrymen of Greater Britain.

---

MANY complaints have from time to time been made of the sanitary condition and drainage of the Adelaide Hospital, and the dangers to patients arising therefrom. The recent report of the Central Board of Health, which states that "all the medical officers agree that during the last ten years there has not been experienced in the wards anything like dangerous "hospitalism" or hospital gangrene, nor frequent cases of pyæmia, blood-poisoning, or erysipelas," is therefore distinctly reassuring. Unfortunately, however, it is certain that the building is not in accordance with the most approved modern principles, and the drainage system is most defective and imperfect. "Judgment and intelligence alike," so runs the criticism of our contemporary, *The Adelaide Observer*, "appear to have been entirely wanting when the ventilation and the drains were planned." We trust the Adelaide authorities may find it possible to infuse both these desirable qualifications into the individuals they select to carry out the necessary alterations.

